# MILD BEHAVIORAL IMPAIRMENT AS A PREDICTOR OF COGNITIVE FUNCTIONING IN OLDER ADULTS

**DOI:** 10.1093/geroni/igz038.1739

**Published:** 2019-11-08

**Authors:** Hillary J Rouse, Brent J Small, John Schinka

**Affiliations:** University of South Florida, Tampa, Florida, United States

## Abstract

Background: Mild behavioral impairment (MBI) is considered to be a late life transitional state between normal aging and dementia that describes individuals who have persistent behavioral changes and/or psychiatric symptoms. Individuals with MBI are found to be at greater risk of dementia compared to those without these symptoms. Identifying how MBI might relate to different domains of cognition is of key importance, as it could be an early indicator of a future dementia diagnoses. Method: Secondary data analysis of a sample (n=512) of older adults from the Florida Alzheimer’s Disease Research Center who were either cognitively healthy or presenting with mild cognitive impairment (MCI). Some individuals presented with MBI, as defined by decreased motivation, affective dysregulation, impulse dyscontrol, social inappropriateness, or abnormal perception/thought content. Executive function, attention, short-term memory, and episodic memory, were compared using a battery of neuropsychological assessments. Results: Individuals with MCI performed worse on all tasks across all cognitive domains, where individuals with MBI performed worse on several tasks associated with executive function, attention, and episodic memory. Compared to individuals with only MCI, individuals with MCI and MBI performed significantly worse on tasks associated with executive function and episodic memory. Conclusion: The present study found evidence that individuals with MBI will perform worse on tasks of executive function, attention, and episodic memory. Further, those with MCI and MBI will perform significantly worse on executive function and episodic memory tasks. Future research should explore if these findings can help to predict specific dementia diagnoses.

